# What happens next in dynamic natural scenes: Temporal discrimination is modulated by motion and scene context but not stereoscopic depth

**DOI:** 10.1167/jov.26.7.8

**Published:** 2026-07-16

**Authors:** Jiali Song, Ginnie Wee, Benjamin Wolfe

**Affiliations:** 1 Department of Psychological & Brain Sciences, University of Toronto Mississauga, Mississauga, ON, Canada

**Keywords:** scene perception, prediction, road scenes, stereoscopic depth, mental representation

## Abstract

We investigated mental representations of real road scenes using a prediction task. On each trial, 48 licensed drivers watched a road scene for 2 seconds and immediately after selected what the scene would look like 2 seconds in the future in a five-alternative forced choice (5AFC) task. We factorially manipulated three visual properties of the preview to examine their effects on performance: motion, scene context, and stereoscopic depth. We developed a binocular dashcam video dataset of real road scenes simulating human interpupillary distance. We manipulated motion by displaying still images or video previews. Scene context was manipulated by showing videos recorded on urban roads, which are visually denser, or in highway roads, which are visually sparser. To investigate whether small disparity signals are used in this task, we manipulated stereoscopic depth by varying stimulus presentation: Each eye received video from the corresponding camera, only one eye received video, or both eyes received identical videos. Participants were able to correctly select the future appearance of the road to some extent, with better precision for video than stills and for urban scenes than highway scenes. Stereoscopic cues did not affect performance, indicating that participants likely relied more heavily on monocular depth cues in this task. These results suggest that mental representations of complex natural scenes are temporally imprecise and are consistent with, but do not uniquely require, projections of future states.

## Introduction

Many daily tasks require an accurate mental representation of our environment. For example, catching a ball requires an accurate understanding of the trajectory of the ball to position the hand at a location that will intercept the ball. Similarly, a driver needs to accurately represent the road environment to anticipate hazards and avoid collisions. An accurate representation of the road scene requires tracking multiple objects on the road, the structure of the road, and self-motion. Conversely, an inaccurate representation can have catastrophic consequences. Driving is a suitable context for investigating the quality of mental representations because road scenes are highly structured spatially and temporally, and they closely mimic our lived environment. These qualities allow observers to make judgments about the road environment using their mental representation of it, which can be assessed for its accuracy.

An accurate mental representation of the road supports anticipation of hazardous road events and timely evasive responses. Under the situation awareness (SA) framework (Endsley, 1995), a popular theoretical framework under which human factor researchers conceptualize driver awareness, the ability to accurately predict and anticipate future states of the environment represents the highest level of awareness. According to this framework, better mental representation should also include better representation of future states of the road.

Although the SA framework does not distinguish among different kinds of future-oriented cognition, we would like to distinguish between anticipation and prediction. We use anticipation to refer to representations of future states revealed by responses to the stimulus at hand, such as positioning the hand to where a projectile will land prior to it reaching there to catch it, moving the eyes ahead of the trajectory of a moving stimulus (Becker & Fuchs, 1985; Westheimer, 1954), or looking at an object ahead of an expected interaction (Eisenberg, Zacks, & Flores, 2018; Hayhoe, Shrivastava, Mruczek, & Pelz, 2003; Land, 2006). Conversely, we use prediction to refer to self-reported future states of the environment, such as the identity of an object that may appear in a certain location, or the likelihood of an event occurring in the immediate future (Szpunar, Spreng, & Schacter, 2014). Given that anticipation does not require conscious report but prediction does require conscious report, the information that enables anticipation may not necessarily be consciously accessible and therefore may not be reportable in a task that probes prediction. As such, tasks that measure anticipation and prediction may not necessarily rely on the same representation or underlying cognitive processes.

In a driving context, evidence of both anticipation and prediction has been reported. Anticipation in driving takes many forms, such as anticipatory looking (He, Kanaan, & Donmez, 2021; Katrahmani, Ahmadi, & Romoser, 2017; Lehtonen, Lappi, Kotkanen, & Summala, 2013; Song, Zivli, & Wolfe, 2026), hovering the foot over the brake in preparation of the onset of a hazard (Muttart, 2003; Yahoodik, 2023), or slowing down in anticipation of a hazard despite the absence of overt visual signals of hazards (latent hazards) (Crundall et al., 2012; Vlakveld et al., 2011). These processes are more similar to the demands a driver faces on the road than predictions, as drivers tend very rarely to report their predictions and instead intend to act appropriately to evade a collision.

Prediction has also been investigated in a driving context. The most common way to index prediction is to ask licensed drivers to describe what happens next in videos with real and staged road hazards (Jackson, Chapman, & Crundall, 2009; Katrahmani et al., 2017), or by choosing the best description out of four options (Kroll et al., 2020; Ventsislavova & Crundall, 2018). It has also been probed by asking participants to draw the trajectory of all road users within 2 seconds after the end of a road video in a top-down video (Vogel, Kircher, Alm, & Nilsson, 2003). Then, videos were coded as correct or incorrect based on whether the participant correctly predicted whether each vehicle yielded to other road users. More correct predictions in these tasks are correlated with lower crash risk (Horswill, Hill, & Jackson, 2020). Moreover, consistent with the idea that anticipation and prediction are different processes, more accurate anticipation responses may not necessarily correspond to more accurate prediction responses (Gillies & Kosovicheva, 2025; Vogel et al., 2003), as drivers may report the correct maneuver even if they were unable to correctly report how a safety critical situation will unfold (Gugliotta et al., 2017).

This study focused on the consciously accessible information that participants can report. Investigating what visual information impacts observers’ ability to make predictions can reveal the basis of these judgments. Many previous studies on prediction using driving-relevant stimuli have used verbal descriptions and probes about behavior of specific road users, but these studies have not been able to assess the mental representation of the whole scene, only what was probed. Furthermore, verbal descriptions are inherently lossy, and there may be information accessible to awareness that is not amenable to verbal report, such as the precise speed or spatial arrangement of objects. Even when trajectories have been reported (Vogel et al., 2003), the analyses conducted were not detailed enough to assess the temporal precision of predictions, which are necessary in order to characterize how these judgments are modulated by the available visual information.

One prior study probed the accuracy of predictions of real road scenes using a two-alternative forced choice (2AFC) temporal discrimination task (Wolfe et al., 2019). Participants previewed a natural road scene and immediately after identified which of two alternative frames was nearer in time to the end of the preview. One of the alternatives was always from 500 ms after the end of the preview, and the other from a later point. By varying the preview duration and the separation in time between the two alternatives, it was found that participants could reliably identify the nearer frame given sufficient preview duration (>0.5 second) and sufficient temporal separation between frames (>0.73 second), although performance increased with longer duration and larger frame separation. These findings are consistent with the idea that observers can use their mental representation to identify the immediate future. However, this task can be done by selecting the frame most similar to the last seen frame, and it is unclear the extent to which the mental representation allows participants to report predictions about a specific temporal horizon.

## Present study

The goal of the present study is to investigate whether participants can predict the future state of dynamic road videos with a specific temporal horizon and the visual information used in making these judgments. Participants were asked to imagine what a road scene would look like 2 seconds after the end of a preview. We used a five-alternative temporal discrimination task to probe a range of times surrounding the target time, so participants could not rely only on image similarity to identify the correct response. This method also allowed us to estimate the precision and accuracy of the judgment over multiple trials. Based on prior literature reviewed above, we expected that participants would perform this task above chance.

We also investigated the effects of three visual factors on prediction accuracy: motion, stereoscopic depth, and scene context. We hypothesized that motion should improve prediction performance compared to static images because videos convey more information about speed of travel than static images, and object speed influences the mental representation of the scene such that scenes depicted as containing faster motion can alter the remembered appearance of the scene (Freyd & Finke, 1985; Hubbard, 2005). Given the range of speeds that vehicles can adopt on the road, there may be significant uncertainty about the speed of the viewpoint vehicle and that of other vehicles on the road in the absence of motion. Therefore, providing more information should improve the accuracy of prediction by allowing a more accurate estimate of speed. If so, prediction performance for video previews should be better than with still images. However, given that implied motion can bias the remembered mental representation of scenes (Freyd & Finke, 1984; Intraub, 2002), still image previews may be less accurate or precise than dynamic previews that provide speed information.

Stereoscopic depth is a visual cue that conveys spatial information about relative distances of objects (Cutting & Vishton, 1995). It is produced by binocular disparity, small differences in the retinal image between the two eyes, a factor seldomly investigated in the context of driving. It is believed that binocular disparity as a depth cue is used at viewing distances up to 5 meters because disparities become very small beyond 5 meters, making it less reliable than other depth cues such as occlusion and linear perspective (Cutting & Vishton, 1995). Because distances relevant for driving are often much farther than 5 meters, it may be reasoned that binocular disparity does not play an important role in the driving context. However, more recent research found that human observers are able to accurately judge depth order from small binocular disparities even at 40 meters away and beyond (Palmisano, Gillam, Govan, Allison, & Harris, 2010), suggesting that such small binocular disparities can be used to infer spatial layout. However, the study was done in a visually impoverished environment (a long dark tunnel) and may not accurately reflect everyday contexts where depth is much more redundantly represented by monocular depth cues. If binocular disparity serves a functional role in a more naturalistic task such as predicting the appearance of road scenes, the addition of stereoscopic depth should improve prediction.

Scene context likely matters for prediction performance, as well, as predictions should reflect expectations about the visual stimulus. One contextual factor particularly relevant to road scenes is the type of road environment. In Wolfe et al. (2019), the accuracy of prediction performance was higher for urban roads compared with highway roads, even across preview duration and frame separation. Wolfe et al. (2019) argued that urban road scenes are more visually dense and complex compared to highways. The increased visual information present in urban road scenes may facilitate scene prediction and make it easier to discriminate between two frames from the same video. Moreover, roads are designed to accommodate traffic at different speeds. For example, highways tend to be wider and have more lanes than urban roads. These aspects of the appearance of roads may set up expectations about travel speed. If expectations can modulate the memory representation of the scene (Reed & Vinson, 1996; Verfaillie & d'Ydewalle, 1991), one may expect expectations about travel speed to also modulate explicit predictions of road scenes. For these reasons, we expect performance to differ between urban and highway roads.

## Methods

### Sample

Forty-eight licensed drivers were recruited to participate in the study from the University of Toronto Department of Psychology Participant Pool ages 18 to 29 years (average age, 19.9 years; *SD* = 2.4 years). One of the 48 licensed drivers declined to complete the survey, so their driving experience is unknown. For the other 47 licensed drivers in the sample, mean driving experience was 3 years with a *SD* of 2.35 years. All participants had near acuities of 20/25 or better in each eye, as confirmed by an Early Treatment Diabetic Retinopathy Study (ETDRS) test at a 40-cm viewing distance, and a minimum stereo acuity of 80 seconds of arc, as confirmed by the Titmus Circle Stereotest. Furthermore, all participants viewed a demonstration video with binocular disparity before the start of all trials, and they were asked whether they perceived it as three-dimensional (3D) and different from the monocular and no-disparity display conditions. One participant was replaced for not perceiving the demonstration video as 3D. A further two participants were replaced due to software error, and one participant was replaced due to refusal to use the chinrest.

We pre-registered that we would replace participants whose overall accuracy was not significantly different from chance using a binomial test; however, 14 out of 48 participants did not meet this criterion, which represents approximately 30% of the sample. At the time of pre-registration, we had underestimated the difficulty of the five-alternative forced choice (5AFC) task despite having piloted the methods, as all pilot participants were able to perform the task significantly above chance. We intended to only exclude participants who failed to follow instructions or understand the task. However, given the difficulty of the task, we recognized that it is possible to perform at near chance levels despite understanding the task. Furthermore, under the consideration that examining the type of errors that participants made was also informative, we decided to only exclude participants who performed below chance. Under the new inclusion criterion, no participants were excluded based on their performance. Further, analyzing the performance of participants regardless of whether their performance was significantly above chance also enabled us to statistically evaluate whether the sample performed differently from chance.

### Equipment

Videos were shown using an OptiPlex 5040 computer (Dell Technologies, Round Rock, TX) with a Quadro M2000 graphics card (NVIDIA, Santa Clara, CA) connected to a UHZ45 Projector (Optoma, New Taipei City, Taiwan) and a 3D polarizer (DepthQ, Bellevue, WA) in a rear-projection configuration on a Stewart Filmscreen 150 3D screen (Stewart Filmscreen, Torrance, CA). Using this set-up, we presented stereoscopic videos at 720-pixel resolution and a refresh rate of 120 Hz using Psychtoolbox (Brainard, 1997; Kleiner et al., 2007) in MATLAB 2023b (MathWorks, 2026). The display size was 93 by 52 cm, and extended 100 × 56 degrees visual angle (dva) from a viewing distance of 39 cm. The video previews were displayed at 78 × 44 dva, and the response choice were displayed at 2.5 times smaller than the preview video, at 42 × 23.9 dva.

### Stimuli

Road videos were captured by author JS in the Greater Toronto Area in Ontario, Canada. To obtain videos with stereoscopic depth information, we set two dash cams (Garmin Dash Cam Mini 2; Garmin, Schaffhausen, Switzerland) near the center of the windshield inside the car. The two cameras were placed horizontally with a fixed lens center distance of 66 mm to approximate human interpupillary distance. Videos were recorded at 1080 pixels and 29.97 Hz. Videos in the two cameras were synced using audio channels and converted to 720 pixels for display. To ensure that the scenes changed sufficiently over the duration of the preview and test period, only portions where the vehicle was moving were used as stimuli. Segments of video where the car was stopped (e.g., at a light or in traffic) or moving extremely slowly (approximately under 20 km/hr) were annotated manually and excluded from the experiment. Furthermore, road type (highway or local road) was also annotated manually. Half of the stimuli were taken on urban roads with speed limits of 50 to 70 km/hr, and half were taken on multiple-lane highways with a speed limit of 100 km/hr. The actual speeds varied but were always at or below the speed limit. All annotations were done by a licensed driver and checked independently by author JS. Finally, all videos were calibrated to converge at 10 meters ahead by shifting the left-eye image leftward until the calibration target (located at 10 meters ahead) converged. This required only minimal correction per video, ranging from 9 to 12 pixels corresponding to 0.5 to 0.7 dva. Examples of Highway and Urban stimuli videos are available as Movie S1, Movie S2, Movie S3, and Movie S4.

### Procedure

On each trial, participants predicted what the scene would look like 2 seconds after the end of a 2-second preview. Each trial began with a fixation point at screen center, followed by a random dot mask of the same dimensions as the preview for 250 ms. Then, participants viewed a preview, which was a 2-second excerpt from a road video. After the end of the preview, a random dot mask appeared for 250 ms. Next, on the response screen, participants viewed five static test images and clicked on the image that best matched their prediction using the computer mouse. The images were arranged in a random order on each trial and were arranged in two rows: three on the top and two on the bottom at a size 2.5 times smaller than the preview video.

**Figure 1.**
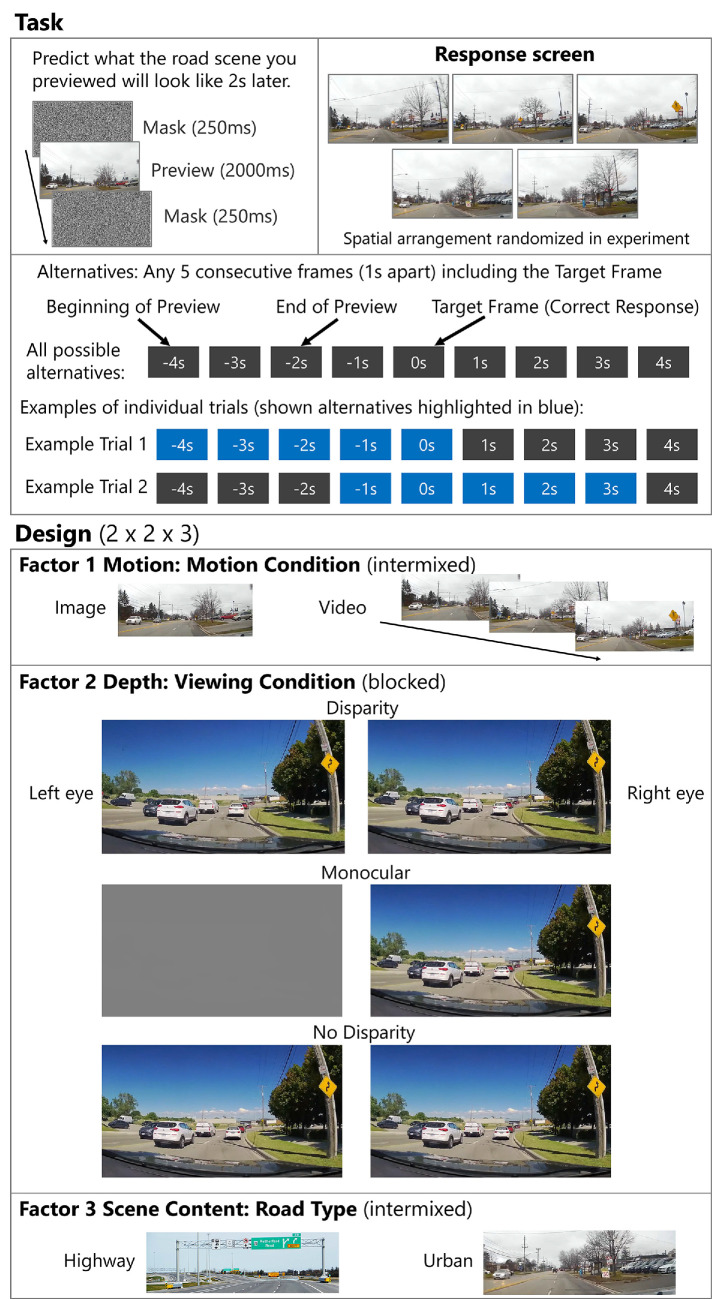
A schematic of the procedure and design of the study. The box with the heading “Alternatives” indicates the pool of possible frames that all test frames can be sampled from. On each trial, five frames that appeared adjacent to each other in the schematic were selected. There were five possible configurations, all of which contained the Target Frame (shown in table format in Supplementary Table S4). Due to this sampling procedure, the Target Frame had equal probability of appearing at any of the five temporal positions on any given trial, including the earliest and latest presented frame. In the Depth manipulation, different images were shown to each eye in the Binocular condition. The example illustrated in this figure could be viewed using parallel fusion. In the No Disparity condition, both eyes viewed the image shown to the right eye in the Binocular condition.

On each trial, the test images were sampled using a sliding window of 5 seconds around the target time at 1-second intervals. The target time refers to +2 seconds from the end of the video preview or from the still preview. Figure 1 shows a schematic of all possible choices that appeared on the response screen throughout the entire study. Test images spanned from –4 seconds relative to the target time (the beginning of the preview video) to 0 seconds at the earliest, and from 0 seconds to 4 seconds relative to the target time at the latest. As a result, all times tested included 1-second intervals that spanned the range of –4 seconds (the beginning of a video preview or 2 seconds before a still preview) to +4 seconds relative to the target time (+2 seconds from the end of the preview). The earliest choice had an equal probability of being –4, –3, –2, –1, or 0 seconds from the target time, such that the window of time tested varied across trials. Each possible 5-second window had an equal number of trials, and the order in which test images were arranged were also randomized on each trial. Following this procedure, the correct frame was on screen every trial, resulting in a chance performance of 20%. Furthermore, of the five test frames shown, the target frame had an equal proportion of trials at each of the five frames, such that arranging the test frames in temporal order and then always choosing the frame that appeared in the middle of the tested interval also would have resulted in a 20% chance of selecting the target frame.

To examine the effect of binocular disparity, we varied the viewing condition in three blocks: binocular viewing with disparity, in which participants viewed the preview from the left camera perspective in the left eye and the right camera perspective in the right eye, such that there was both binocular disparity and disparity-defined depth in the stimuli; monocular viewing, in which participants viewed the footage of the right camera in the right eye and a gray screen (the background color) in the left eye, such that there was disparity between the eyes but no disparity-defined depth information present in the stimuli; and binocular viewing, in which participants viewed footage from the right camera perspective in both eyes (similar to two-dimensional displays), such that there was no binocular disparity in the videos and also no disparity-defined depth information. These three viewing conditions were completed in blocks, the order of which was counterbalanced across participants.

To examine the contribution of motion on prediction performance, previews were either a video or a still image, and they occurred in equal proportions, randomly intermixed throughout the experiment. Still images were taken from the end of what would have been a video preview such that the target for the corresponding video and the still images were identical. Half of all videos depicted urban roads and the rest depicted highways, and these were also intermixed throughout the experiment.

There were 20 practice trials at the beginning of each block to familiarize participants with the viewing condition, followed by 120 experimental trials. All combinations of motion and road types were presented in equal proportions in the practice trials. Participants received feedback about the accuracy of their responses during the practice trials, where the correct frames were indicated to them with a red outline. There was no feedback present in the experimental trials. In total, the entire experiment had 420 trials, 360 of which were experimental trials that were included in the analysis. Participants were given the opportunity to take a break every 80 trials, and the experiment typically lasted 1.5 hours.

### Data analysis

We pre-registered several dependent variables to measure prediction performance. We examined the proportion correct of the selected frame to determine the extent to which participants could accurately predict the appearance of the scene. We also examined time error, defined as the difference between the time of the selected frame and the target time. We also examined the standard deviations of time errors as a measure of how consistent and precise predictions were across trials.

To identify systematic bias toward the future in participants’ predictions, we examined whether time error was significantly larger than 0 using a one-sample, one-tailed *t*-test. This was a pre-registered analysis. We pre-registered separate analyses of variance (ANOVAs) investigating how preview condition interacted with viewing condition and road type for each dependent measure. The pre-registered analyses were analogous to the simple main effects analyses of two-way interactions in a three-factorial design. However, in the pre-registration, we did not account for a three-way interaction among the three factors manipulated. To account for possible three-way interactions, we deviated from the pre-registration by conducting three-way within-subjects 2 (preview condition: still and video) × 3 (viewing condition: binocular, monocular, and no disparity) × 2 (road type: urban and highway) omnibus ANOVAs separately on each of the dependent measures described above. The full omnibus ANOVAs encompassed the pre-registered tests to test specific hypotheses, and all results presented here were interpreted in the context of the omnibus ANOVA.

To investigate the relation between reaction time (RT) and prediction performance, we also examined whether participant-wise RT was associated with accuracy, time error, and the time error standard deviation. These exploratory analyses were not pre-registered. All effect sizes reported here are generalized eta squared ($\eta_{g}^{2}$) (Olejnik & Algina, 2003).

## Results

### Proportion correct

**Figure 2.**
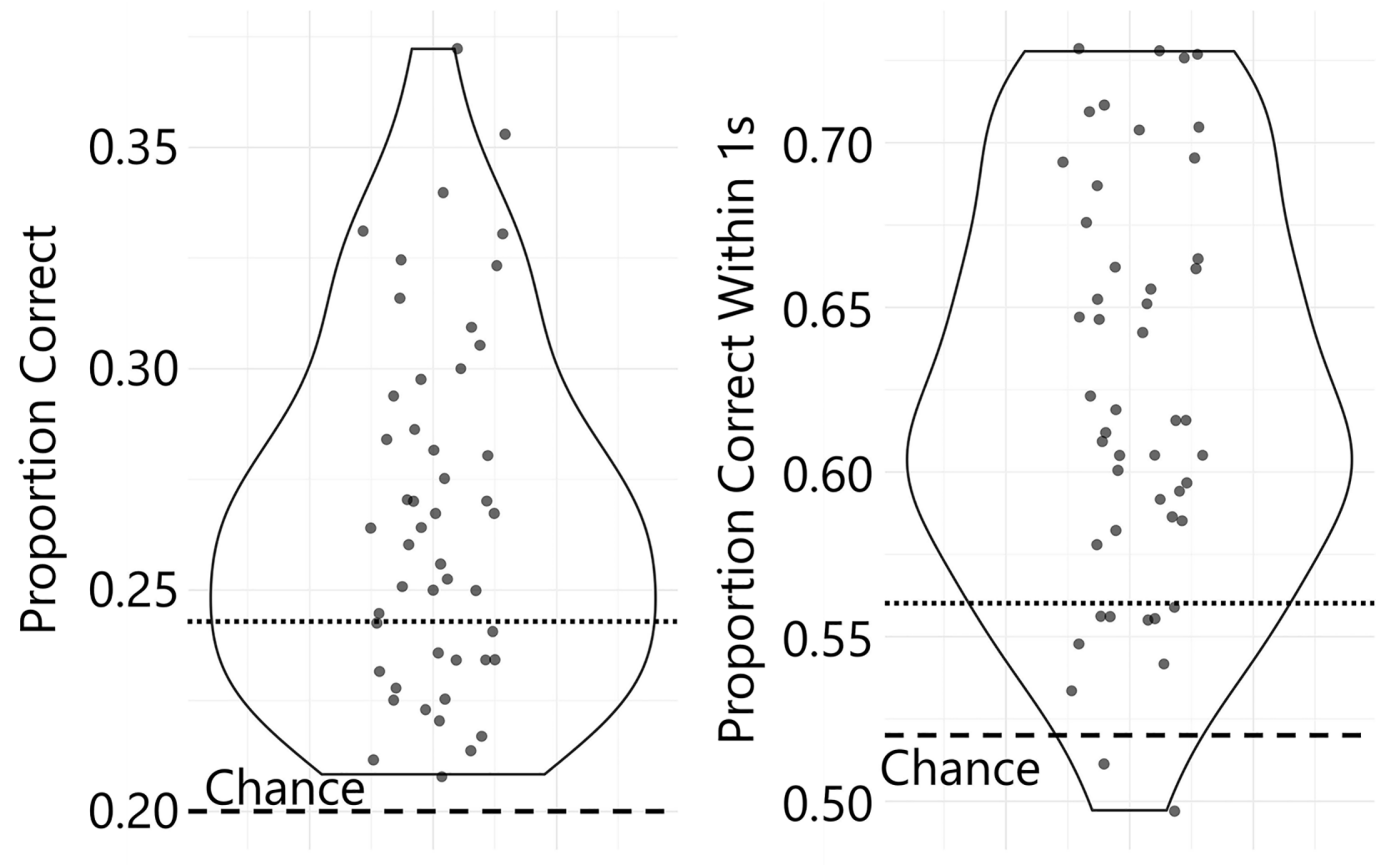
Violin plots of overall performance in the prediction task across all conditions. The left panel shows the overall proportion of selecting the correct target frame. The right panel shows overall accuracy if the target frame and ± 1 second were considered correct. Figure conventions were identical for both panels. Each circle represents a single participant. The dashed horizontal lines represent chance performance, and the dotted horizontal lines represent the critical accuracy required for a significant one-way binomial test against chance performance. Note that the two panels have different *y*-axes.

Overall, performance for all participants was above chance numerically (*M* = 0.27, *SD* = 0.04), but 14 out of 48 participants did not perform statistically significantly greater than chance according to a one-tailed binomial test. These results suggest that the task was quite difficult. However, if predictions were correct but imprecise, we could expand what we considered to be correct, including frames 1 second before and 1 second later relative to the target frame, and accuracy should increase. Under this more relaxed categorization (*M* = 0.62, *SD* = 0.06), 10 participants’ performance was not significantly greater than chance, which is consistent with the idea that predictions were imprecise. Figure 2 shows each participant's overall accuracy according to these two schemes in two separate violin plots.

**Figure 3.**
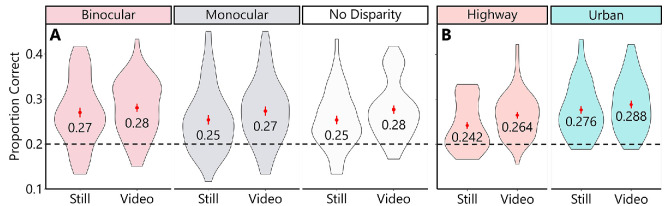
Violin plots showing proportion correct of responses. (**A**) Proportion correct as a function of preview condition and viewing condition, collapsed across movie type. (**B**) Proportion correct as a function of preview condition and movie type, collapsed across viewing conditions. Figures with data in the fully expanded 12 unique conditions are available in the Supplementary Materials. Figure conventions were identical for both panels. Red dots represent the mean, and red vertical lines represent the standard error of the mean (*SEM)*. The dashed horizontal line represents chance performance.

Figure 3 ^4^A shows the proportion correct as a function of preview condition and viewing condition collapsed across road types, and Figure 3B shows the proportion correct as a function of preview condition and road type, collapsed across viewing conditions. Because we found no significant two-way interaction between viewing condition and road type, the figures provided here have been collapsed for legibility. Figures showing the data in all 12 fully crossed conditions are available in the Supplementary Materials.

**Figure 4.**
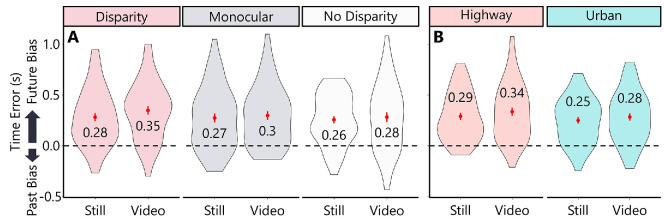
Violin plots showing the average time error per participant and condition, defined as the difference in time between the target frame and the frame selected by the participant. (**A**) Time error as a function of preview condition and viewing condition, collapsed across movie type. (**B**) Time error as a function of preview condition and movie type, collapsed across viewing conditions. Figure conventions are the same as those of Figure 3, except positive values above the dashed horizontal line represent bias toward the future, where participants responded further along in time compared with the target. Negative values below the dashed horizontal line represent biases toward the past, where participants selected earlier times relative to the target. Overall, participant selections tended to be biased toward the future, and the bias was significantly greater than 0.

The 2 × 3 × 2 omnibus ANOVA on proportion correct found a significant main effect of preview condition, *F*(1, 47) = 9.41, *p* = 0.003, $\eta_{g}^{2}$ = 0.01), such that the proportion correct was significantly higher for video previews (*M* = 0.28, *SD* = 0.09) than for still previews (*M* = 0.26, *SD* = 0.09). This effect was small, as the 2% difference between preview conditions corresponds to approximately three trials, but was consistent across participants and conditions (see Supplementary Materials for subject-level data). There was also a significant main effect of road type, *F*(1, 47) = 20.5, *p* < 0.001, $\eta_{g}^{2}$ = 0.03), such that the proportion correct was significantly higher for urban (*M* = 0.28, *SD* = 0.09 ) than highway (*M* = 0.25, *SD* = 0.09) videos. This effect was also small, corresponding to a difference of approximately five trials. No other effects were significant, including any effects involving viewing condition (*F* < 0.09, *p* > 0.4, $\eta_{g}^{2}$ < 0.003; see Supplementary Materials for a full statistics table).

### Mean time error

We examined mean time error as a measure of overall magnitude of prediction error as shown in Figure 4. Positive time error represents a future bias, in which participants tend to respond further along in time than the correct frame, whereas negative time error represents a past bias in which participant responses are not as far along in time as the target frame. The one-tailed one-sample *t*-test on average time error found that the average time error was significantly greater than 0, *t*(47) = 9.39, *p* < 0.001, *M* = 0.29, *SD* = 0.21, indicating that there was an overall bias toward the future.

The 2 × 3 × 2 omnibus ANOVA found a significant main effect of road type, *F*(1, 47) = 4.79, *p* = 0.03, $\eta_{g}^{2}$ = 0.004), such that the time error was slightly larger for the highway (*M* = 0.31, *SD* = 0.34) than urban (*M* = 0.27, *SD* = 0.34) videos. There was no significant main effect of preview condition, *F*(1, 47) = 2.8, *p* = 0.10, $\eta_{g}^{2}$ = 0.003, even though the magnitude of time error was numerically higher for video previews than still previews. There was also no significant main effect of viewing condition, *F*(2, 94) = 0.82, *p* = 0.44, $\eta_{g}^{2}$ = 0.003, nor any other effects (*F* < 1.16, *p* > 0.31, $\eta_{g}^{2}$ < 0.002; see Supplementary Materials for a full statistics table).

### Time error standard deviation

**Figure 5.**
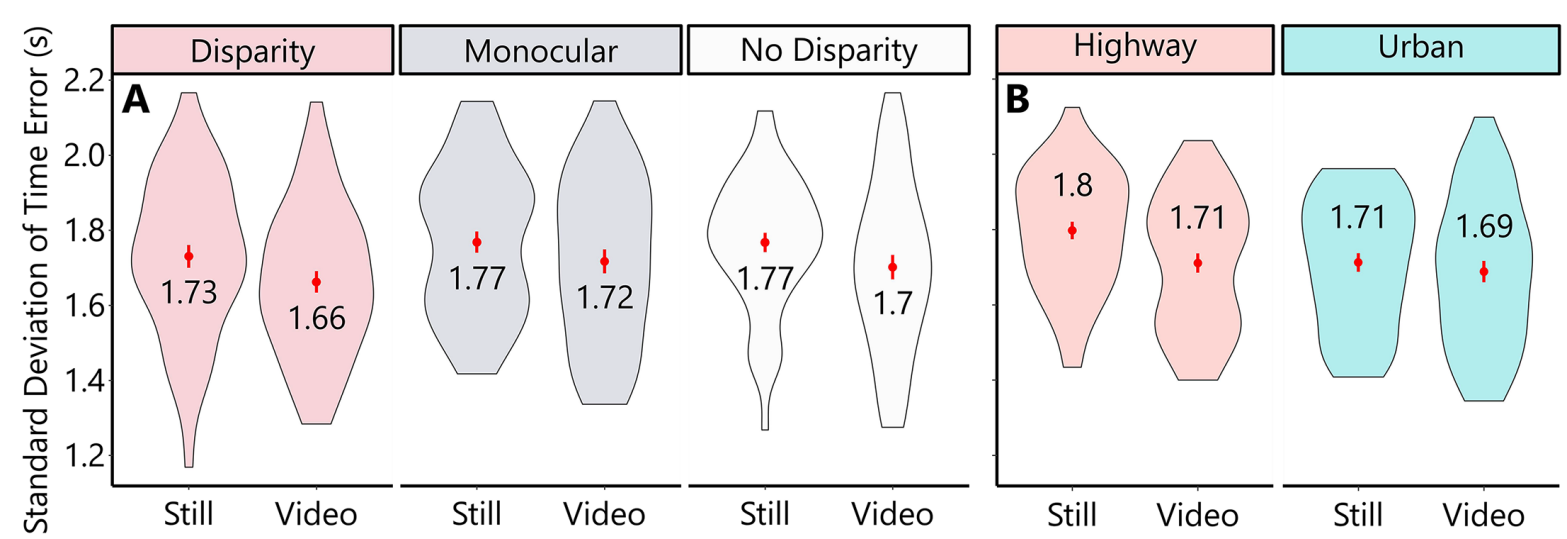
Violin plots of the standard deviation of time error per participant and condition. (**A**) Time error standard deviation as a function of preview condition and viewing condition, collapsed across movie type. (**B**) Time error standard deviation as a function of preview condition and movie type, collapsed across viewing conditions. The time error standard deviation is a measure of within-participant consistency, such that the lower the standard deviation, the more a participant tended to select the same frame across trials, regardless of whether the selected frame was the target frame. Figure conventions are otherwise identical to those of Figure 3.

Figure 5 shows the within-participant standard deviations of time error, which is a measure of how consistent a participant is across trials and, by extension, the precision of their responses. For example, a participant who always selects the frame +2 seconds from the target will have a lower standard deviation than a participant who selects the target frame and the +1 second frame approximately equally, even though the latter participant selects the correct response more often.

**Figure 6.**
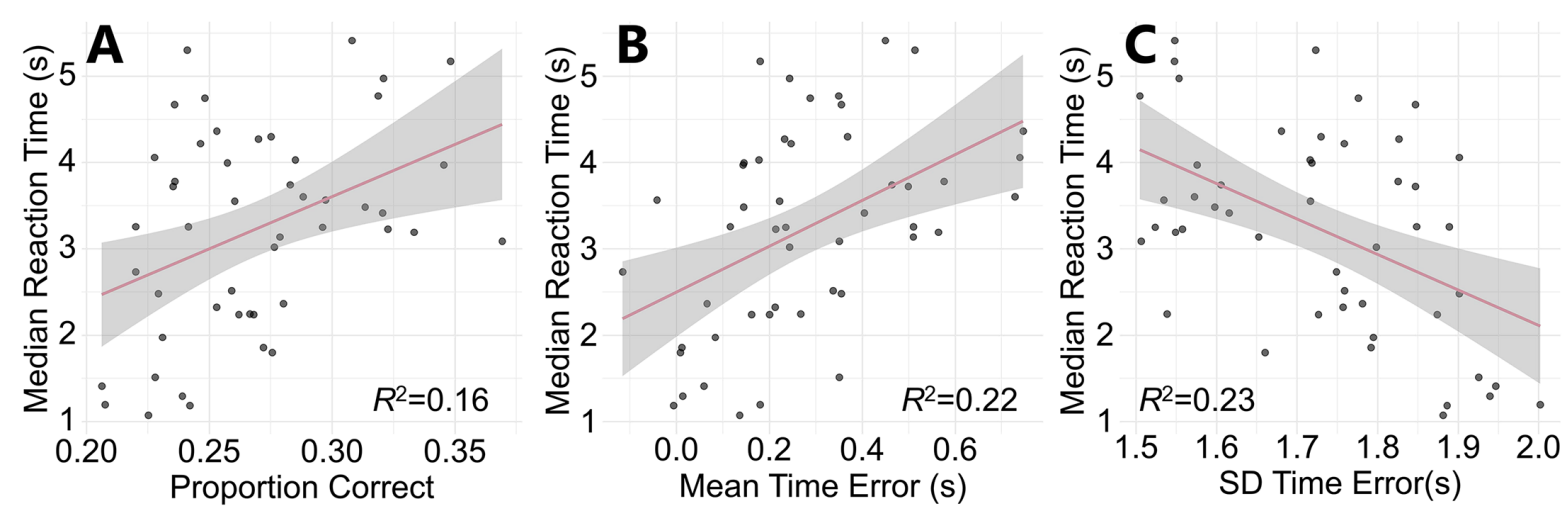
The correlation between median RT for each participant and their performance in the prediction task. (**A**–**C**) Relation between median RT and proportion correct, mean time error, and time error *SD*, respectively. Figure conventions are identical in all three panels. Each dot represents a single participant, the dark pink line represents the regression line fit using a linear model, and the shaded gray area represents the 95% confidence interval of the regression line.

The 2 × 3 × 2 omnibus ANOVA on the time error standard deviation found a significant main effect of preview condition, *F*(1, 47) = 10.43, *p* = 0.002, $\eta_{g}^{2}$ = 0.01, such that the standard deviations were lower for video previews (*M* = 1.69, *SD* = 0.26) than still previews (*M* = 1.75, *SD* = 0.26). There was a significant main effect of road type, *F*(1, 47) = 9.83, *p* = 0.003, $\eta_{g}^{2}$ = 0.02; the standard deviation was higher for highways (*M* = 1.75, *SD* = 0.26) than urban roads (*M* = 1.69, *SD* = 0.26). There also was a two-way interaction between preview condition and road type, *F*(1, 47) = 5.65, *p* = 0.02, $\eta_{g}^{2}$ = 0.005, such that the effect of preview condition was larger for highway than urban road videos, although the direction of the effect was the same in both conditions. A simple main effects analysis showed that the effect of preview condition was statistically significant for highway videos, *F*(1, 47) = 14.5, *p* < 0.001, $\eta_{g}^{2}$ = 0.07, but the effect of preview condition did not reach statistical significance for urban videos, *F*(1, 47) = 0.88, *p* = 0.35, $\eta_{g}^{2}$ = 0.003, although the effects were in the same direction for both road types.

### Correlation with RT

Next, we investigated the relation between RT and prediction performance by quantifying Pearson's correlation coefficient between RT and proportion correct, mean time error, and the time error standard deviation of participant responses, as shown in Figure 6. There was a significant positive correlation between proportion correct and median RT, Pearson's *r* = 0.40, *t*(46) = 3.00, *p* = 0.004, suggesting a time–accuracy trade-off. There also was a significant positive correlation between median RT and mean time error, Pearson's *r* = 0.46, *t*(46) = 3.55, *p* < 0.001, and a significant negative correlation between median RT and time error standard deviation, Pearson's *r* = –0.49, *t*(46) = 3.79, *p* < 0.001. These results indicate that participants who took longer to respond were more biased toward the future and more consistent across trials.

## Discussion

In this study, we examined the extent to which participants could predict the appearance of natural road scenes 2 seconds into the future using a 5AFC temporal discrimination task as a probe of their overall representation of this natural scene. Our data suggest that mental representations of the road scene included some information about future states or allowed some projection to the future, based on the participants’ ability to perform above chance in this task. However, these predictions were imprecise, as participants often selected frames 1 second before or after the “correct” (target) frame. We also manipulated the availability of motion, scene context, and binocular disparity to examine the visual information that aids prediction performance. We found that motion and urban road contexts improved overall performance by increasing the precision of the chosen frame, indicating that participants relied on motion and scene context to make their predictions. However, performance did not significantly differ among stereoscopic viewing conditions, suggesting that stereoscopic information may not be useful for this task.

Overall proportion correct was low but above chance, suggesting that our temporal discrimination task is quite difficult. When we adopted a more relaxed criterion for the correct response, proportion correct improved drastically for most participants, suggesting that the prediction of entire scenes is possible but with less than 1 second precision for a future state. One effect that contributes to this imprecision is a forward bias: Participants tended to select a frame further ahead in time than the target frame. The average time error of selections was 0.29 seconds, suggesting that participants tended to underestimate 2.39 seconds to be 2 seconds. As the preview did not remain on screen during selection, the predictions had to be based on participants’ memory of the preview. If memory of the last seen frame was subject to a forward bias immediately after preview due to representational momentum, then such forward bias may also propagate to decisions based on memory, including the predicted frame. Although it is difficult to directly compare the magnitude of this forward bias with forward biases observed in the context of representational momentum, the fact that the bias is a fraction of a second is consistent with most representational momentum studies (for a review, see Hubbard, 2005).

### What visual information is helpful for predicting road appearance?

Overall, these data suggest that participants have some awareness of what a road scene would look like in the next 2 seconds. What visual information affects performance? To answer this question, we orthogonally manipulated three aspects of the preview videos: motion by presenting still image or video previews, scene context by presenting urban and highway roads, and stereoscopic depth by manipulating binocular disparity.

#### Motion

We manipulated the available motion information by presenting still image previews with only implied motion and video previews with explicit motion. Our analyses found that the addition of motion information increased the overall proportion correct, which is mainly attributable to an increase in response precision (i.e., lower standard deviations across trials). These results suggest that the presence of motion information improves performance, likely by providing information about distance traveled in 2 seconds which provides more detail on the distance traveled and, consequently, the location of the new viewpoint. These results suggest that motion information is useful for this temporal discrimination task. The magnitude of future bias was not affected by motion, suggesting that motion information itself was not the cause of the forward bias. This is consistent with the idea that the forward bias is driven by representational momentum, as representational momentum also occurs for still images with implied motion (Freyd & Finke, 1984; Intraub, 2002). This suggests that such forward bias may be due to biased memory of the preview and may reflect prior knowledge about the driving context, such as expectation for forward travel.

#### Scene context

To examine the impact of scene context, we manipulated road type: Half of the videos were taken on urban roads and half were taken on multi-lane highways. Overall, our results suggest that urban scenes were easier to predict than highway scenes, as the proportion correct was higher for urban videos than highway videos. This result is well aligned with prior research that found better prediction performance for urban than highway videos (Wolfe et al., 2019). Consistency was also higher for urban videos compared with highway videos, but this was qualified by an interaction between motion and road type. Removing motion information reduced consistency for highway videos but not for urban videos. This may be due to the lower visual density and lack of proximal landmarks in highway videos, which may have made it more difficult to judge speed and distance traveled, particularly when there was no explicit motion information available.

There was also higher time error for highway compared to urban videos, suggesting that participants tended to overestimate distance traveled in 2 seconds more for the highway videos compared with the urban videos. Interestingly, this effect did not vary with motion information, suggesting that even explicitly providing speed information failed to mitigate this overestimation for highway videos. It is unclear why this would occur regardless of the availability of motion. It is possible that an expectation for traveling at high speeds in highway scenarios may have biased speed estimation, particularly in visually sparse environments when there were relatively few cues indicating speed of self-motion compared with the urban videos, which would have resulted in a higher time error for the highway videos. Furthermore, observers have a tendency to underestimate headways (Risto & Martens, 2013; Taieb-Maimon, 2007), and the visual sparsity of highway scenes may have exacerbated these biases because the objects in highway scenes tend to be farther away from the viewpoint than urban scenes.

An alternative interpretation of these results suggested by the effect of scene context is that the participants did not base their selections on the entire scene as a whole but rather relied on one landmark object or a group of specific landmark objects in the scene. If this is true, then prediction performance based on those specific objects alone should be similar to those observed here. A relevant set of questions for future research in scene representation would be what those objects are, how they are monitored, how that information is used, and whether the mental representation of them is more detailed than other parts of the scene that do not affect prediction.

#### Stereoscopic depth

We manipulated stereoscopic depth by presenting a slightly different viewpoint to each eye in the binocular block, by showing only the preview in the right eye in the monocular block, and by showing the same video footage to both eyes in the no disparity block. The results provided no evidence that performance differed among the three blocks for all dependent measures, even though we confirmed that participants had stereoacuities of at least 0.8 arcmin, and participants reported being able to perceive depth in the 3D display while viewing a demo video before the beginning of the experiment.

One possibility is that participants did not need stereoscopic depth cues to estimate distance because there were other depth cues also available. The visual system uses many redundant cues for depth aside from binocular disparity, such as linear perspective, familiar size, and motion parallax, which are combined to form a depth estimate (Landy, Maloney, Johnston, & Young, 1995; Schrater & Kersten, 2000). For the distances involved in road scenes, from a driver's perspective, stereoscopic disparities may be too small and unreliable to be useful, particularly when other more reliable depth cues are available, as depth cue combination is nearly optimal (Harwerth, Moeller, & Wensveen, 1998; Landy et al., 1995). Binocular disparity may be more useful in more impoverished visual environments, such as in long dark tunnels and at nighttime when there are fewer monocular depth cues visible. However, more recent evidence suggests that the depth of 3D shapes is overestimated when more depth cues are available in more complex artificial environments such as in virtual reality (Campagnoli, Hung, & Domini, 2022). Although the disparities involved are much larger than in the current study, it is possible that a more direct measure of distance estimation may be more sensitive to binocular disparity cues compared to the current temporal discrimination task, for which distance estimation is only one possible strategy participants may have employed to accomplish the task.

### Was representation of future states required to perform this task?

So far, the results have been discussed with the assumption that performance in this temporal discrimination task reflected a mental representation of the future state of the scene. However, conceivably, participants could have completed this task without such future representations. We now consider alternative strategies that participants may have adopted to complete this task and whether they are consistent with the data we obtained.

In our analysis of RT, we found a speed–accuracy trade-off: Slower participants tended to be more often correct and more consistent than participants who responded more quickly. These data suggest that taking longer to select a frame is advantageous, but slower participants also tended to have larger biases toward the future than the faster participants. This suggests that strategy use may have affected the results. Furthermore, participants were not likely to rely on these strategies for safe driving, as road situations evolve too quickly to accommodate these long RTs.

One strategy that would reduce average RT is to guess whenever uncertainty is sufficiently high. In aggregate, guesses would produce low consistency and average time errors of approximately zero, and the presence of more guesses would reduce time error and increase the standard deviation. This observed pattern of results is consistent with this idea, suggesting that participants who responded more quickly also gave up earlier and resorted to random guessing more often. Conversely, slower participants may have taken longer to examine each choice during the response screen and been able to use more of the information available to select the correct frame.

One aspect that may explain the overall difficulty of the task is that the test frames can look quite similar even at 1 second apart. This is particularly true for highway videos in which the road scenes tend to be visually sparse. This can lead to participants being unable to discriminate among choices, which decreases their ability to report their prediction. This may also explain the main effect of road type observed in this study, as the higher similarity among choices would make highway trials more difficult than urban trials. These results also suggest that road environments are relatively stable on a second-to-second basis during normal, mundane driving situations in the absence of hazards or high traffic volume, which would not require representation at a finer scale than 1 second. If participants were more able to discriminate the tested frames, then it would be easier to temporally order the test frames and select the correct time. However, more discriminable test frames also make it easier to reject scenes that look dissimilar to the imagined scene, making the current data unable to conclusively reject this strategy.

One strategy is that participants may avoid selecting previously seen frames while randomly selecting from the unseen frames. This would require no representation of unseen future states of the scene, only an accurate memory representation of what was seen. If this strategy of avoiding already seen frames was adopted strictly, the observed overall proportion correct would be 0.26 for video previews and 0.23 for still image previews, consistent with the observed effect of motion. Furthermore, the strategy is consistent with an observed forward bias in time error, but it is more consistent with results in the video preview condition and less likely to explain performance in the still image preview condition because even frames that come from the past relative to the still image preview were unseen. Adopting such a strategy would result in a mean time error of 0.84 seconds in the video preview condition and 0.27 seconds in the still image condition, a much more dramatic effect of motion than the non-significant motion effect on time error that we observed.

One may argue that participants can infer which frames were in the past by ordering the five alternatives, in which case the mean time error of 0.84 seconds would be applicable to all trials. Even so, a time error of 0.84 seconds is significantly larger from the observed time error of 0.29 seconds based on a two-tailed one-sample *t*-test, *t*(47) = –17.83, *p* < 0.001. This smaller time error may be because participants were not able to perfectly avoid past frames (see Supplementary Figure S4 for the distribution of selected frames). This is likely because their representation of the road scene is not precise enough to discriminate among frames for every instance of previously seen frames.

Additionally, this strategy alone also cannot account for the observed standard deviation of time error which is statistically significantly smaller than such a strategy would predict (see Supplementary Materials for more details), in line with the idea that participants have some awareness of the timing of frames that were not previously seen. Even so, the current data cannot conclusively eliminate the possibility that rejecting previously seen frames may have contributed to the observed forward bias, and it is possible that some participants adopted this strategy and others did not.

All of these strategies are consistent with the observed correlation between higher accuracy and slower RT. More time spent on inspecting frames may allow participants to more finely discriminate among frames and thereby do a better job of recognizing and subsequently rejecting previously seen frames, or more accurately determine their temporal order. As such, these data cannot conclusively reject any of these strategies.

The individual variability that allowed us to detect this correlation suggests that different participants may have used different strategies. It may be of interest to examine the extent to which individual participants adhere to each strategy. However, the differences between each strategy are subtle for the dependent measures we examined, and it would be difficult to identify which strategy each participant used. Furthermore, more detailed modeling of these strategies requires new data about how well the participant remembered each preview and how sensitive participants are to differences among alternatives, which may be fruitful future directions to reveal the processes underlying these judgments. Additionally, a single participant may engage in multiple strategies, which would limit the usefulness of this type of individual differences approach and require trial-by-trial analysis.

### Limitations and future directions

One limitation of the study is that this experiment tested only mundane driving scenes where there were no hazardous events. During hazardous events, road conditions and the behavior of other road users may change at a finer temporal scale (i.e., a fraction of a second), which would make such an imprecise temporal representation disadvantageous. However, given the more covert and less active predictions that drivers likely perform on the road, it is unclear whether these covert predictions and anticipatory actions rely on similar sensory inputs as those probed in the current task. It is therefore of interest to examine the extent to which observers can make similar predictions about scenes with the potential for and including unfolding hazardous events, particularly as the ability to report what happens next in these situations has been correlated with driving experience and on-road collision risk (Horswill et al., 2020). We are currently investigating the accuracy of predictions in the context of hazardous road situations.

Finally, stimuli were recorded in the same geographical region as participant recruitment. Consequently, participants may have been familiar with some proportion of the videos that took place in a location that the participants frequent (such as around the university campus). If the spatial layout of the environment is familiar, prediction responses may be more accurate than if the road is unfamiliar. Future work can evaluate the impact of familiarity with the specific layout of the road environment and compare it with the impact of familiarity with the driving situation in general to identify the contribution of different types of experience in forming judgments about the future appearance of natural scenes.

## Conclusions

In summary, participants could, to some extent, identify the future appearance of the road scene 2 seconds ahead, although our data suggest that these temporal mental representations are imprecise. Furthermore, explicit motion and urban road environments improved accuracy and precision of the selection, but the size of these effects was quite small, suggesting that these judgments were based on the available visual information. When this information was comparatively sparse (i.e., in highway scenes), this task became markedly more difficult, suggesting a less precise representation for sparser scenes, although these results may also be due to it being more difficult to discriminate among frames separated by 1 second for sparser scenes. Although stereoscopic depth cues may contribute spatial information relevant to understand the spatial layout of a scene, they did not significantly impact performance, suggesting that observers relied on other sources of depth information, such as monocular depth cues, for this particular task. Stereoscopic depth cues may be more relevant in tasks that require distance judgements, or at nearer distances than those commonly encountered in the scenes we used (e.g., in on-road environments). Overall, these results align with the idea that we build mental representations of complex natural scenes that allow us to make coarse judgments about what the scene will look like in the future, although projection of future states is not uniquely required to make these judgments. Furthermore, these judgments can be modulated by the available motion and spatial information.

## Supplementary Material

Supplement 1

Supplement 2

Supplement 3

Supplement 4

Supplement 5
