# Supplementary material for: What happens next in dynamic natural scenes: Temporal discrimination is modulated by motion and scene context but not stereoscopic depth
Source: J Vis. 2026 Jul 16;26(7):8. doi: 10.1167/jov.26.7.8 (PMC13387269; doi:10.1167/jov.26.7.8)
Supplement: Supplement 1 [file jovi-26-7-8_s001.pdf]

Supplementary Materials for Prediction of natural road scenes: Effects of motion, stereoscopic depth, and scene context

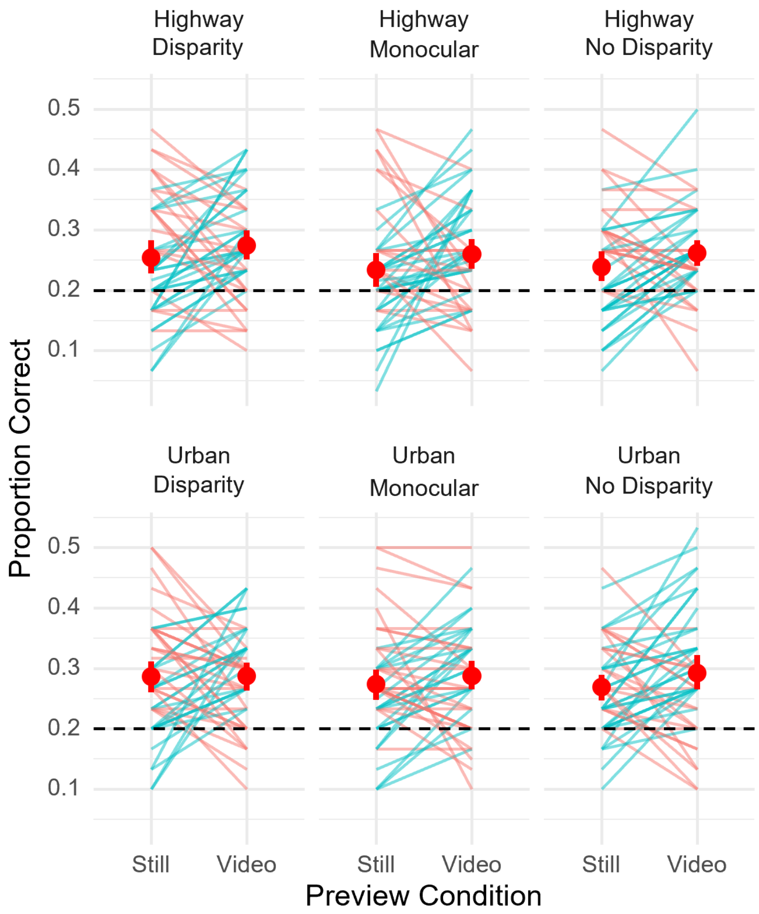

Supplementary Figure 1. Proportion correct of predictions as a function of all Motion Conditions tested in the experiment. Red dots and error bars indicate the mean and SEM, and the colored solid lines represent individual data. Coral represents participants with 0 or negative slopes and turquoise represents participants with positive slopes, meaning that performance was better in Video than Still previews. The black dashed horizontal lines represent chance performance.

**Effect**	***df* 1**	***df* 2**	***F***	***p***	η^2_g
Intercept	1	47	2083.63	<0.001	0.90
Motion Condition	1	47	9.41	0.004	0.01
Disparity Condition	2	94	0.87	0.42	0.003
Road Type	1	47	20.5	<0.001	0.027
Motion Condition × Disparity Condition	2	94	0.30	0.74	<0.001
Motion Condition × Road Type	1	47	0.65	0.42	<0.001
Disparity Condition × Road Type	2	94	0.21	0.81	<0.001
Motion Condition × Disparity Condition × Road Type	2	94	0.18	0.83	<0.001

Supplementary Table 1. Results of omnibus ANOVA on proportion correct.

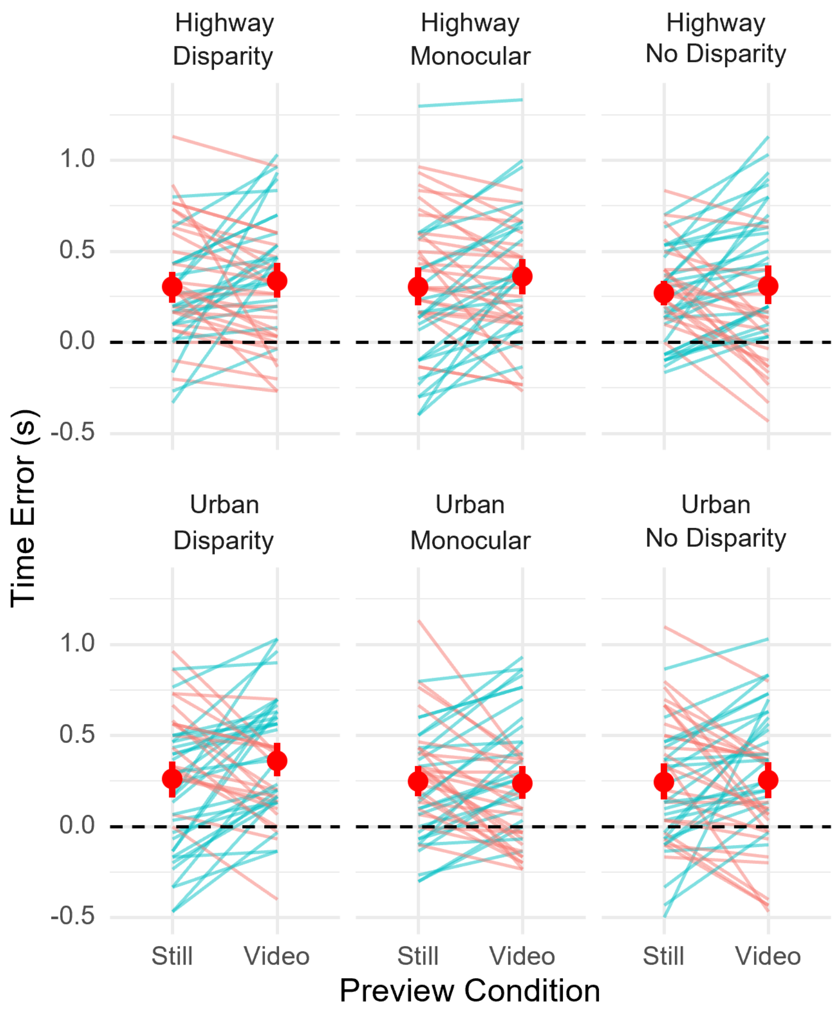

Supplementary Figure 2. Average time error in seconds as a function of all Motion Conditions. Larger magnitudes indicate more time error, with positive errors indicating a bias towards the future, and negative errors indicating a bias towards the past. Figure convention are identical to Supplementary Figure 1.

**Effect**	***df* 1**	***df* 2**	***F***	***p***	η^2_g
Intercept	1	47	88.10	<0.001	0.43
Motion Condition	1	47	2.80	0.10	0.003
Disparity Condition	2	94	0.82	0.44	0.003
Road Type	1	47	4.79	0.03	0.005
Motion Condition × Disparity Condition	2	94	0.37	0.69	<0.001
Motion Condition × Road Type	1	47	0.08	0.78	<0.001
Disparity Condition × Road Type	2	94	1.17	0.32	0.002
Motion Condition × Disparity Condition × Road Type	2	94	1.14	0.32	0.002

Supplementary Table 2. Results of omnibus ANOVA on average time error.

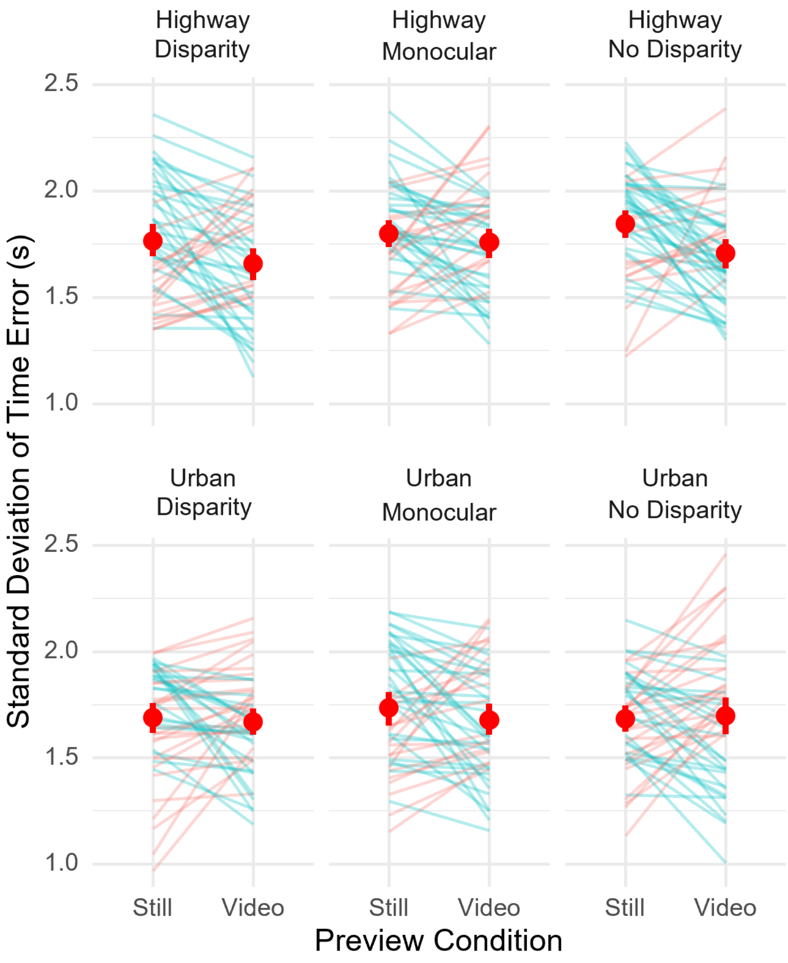

Supplementary Figure 3. Standard deviation of time error in seconds as a function of all Motion Conditions. Lower standard error indicate more consistent responses across trials. Figure convention are identical to Supplementary Figure 1 and 2.

**Effect**	***df* 1**	***df* 2**	***F***	***p***	η^2_g
Intercept	1	47	6338.18	<0.001	0.98
Motion Condition	1	47	10.43	0.002	0.013
Disparity Condition	2	94	2.55	0.008	0.007
Road Type	1	47	9.83	0.003	0.015
Motion Condition × Disparity Condition	2	94	0.06	0.94	<0.001
Motion Condition × Road Type	1	47	5.65	0.02	0.005
Disparity Condition × Road Type	2	94	0.83	0.44	0.002
Motion Condition × Disparity Condition × Road Type	2	94	1.76	0.18	0.005

Supplementary Table 3. Results of omnibus ANOVA on standard deviation of time error.

On the strategy of avoiding previously seen frames

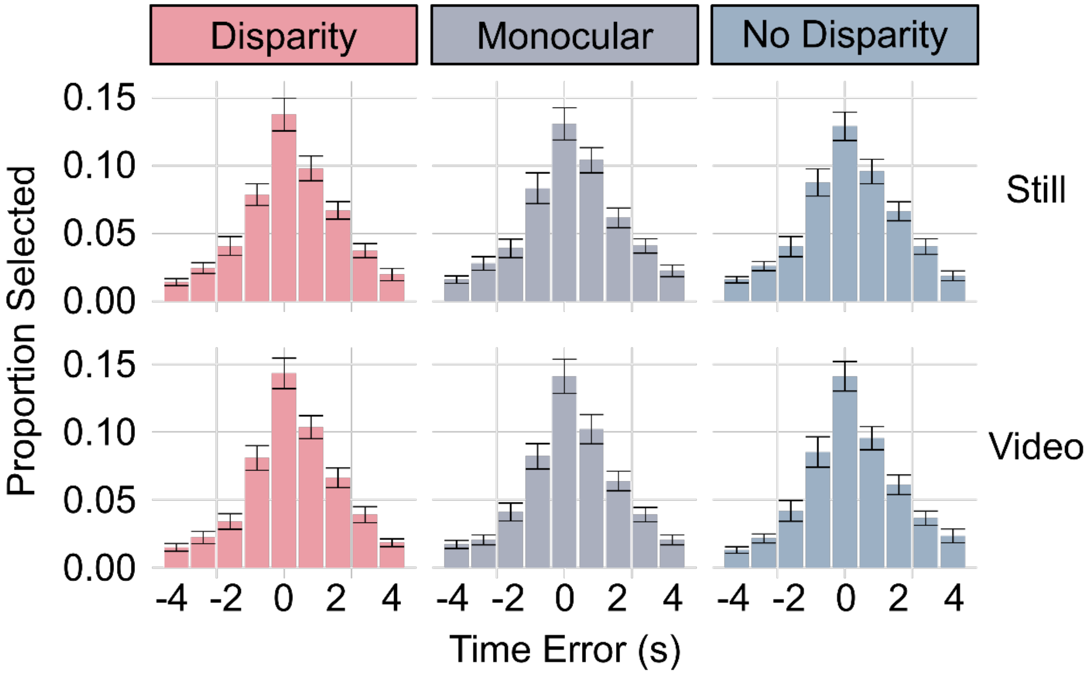

Supplementary Figure 4. Proportion of trials on which a frame was selected across binocular viewing condition and road type. Overall proportions show a positive skew in time errors. Time Errors of -4, -3, and -2 were included in the preview with -2 being the last frame of the preview. Visual inspection indicates that these frames were indeed chosen less often than future frames suggesting that participants could have recognized and avoided choosing already seen frames. However, across all conditions, the +1s frame is consistently chosen more often than the -1s frame. This evidence of forward bias exists even though both -1 and 1 frames are not previously seen, suggesting that the strategy of avoiding familiar frames contributed to the observed forward bias, but could not fully explain the observed data.

Statistical analysis

If participants did not engage in any prediction and picked randomly while perfectly avoiding past frames, then we would expect participants average time error to reflect randomly choosing among frames that they did not previously see. We calculated the mean time error under this strategy by average the time error among only the novel frames, randomly guessing would result in equal probability of each new framed being chosen. The table below shows all possible frames in all trials, which occurs with equal proportion. The time error that should be observed using this strategy would be the mean of the bolded times below, which is equal to 0.84.

To determined whether the observed mean time errors differs from this theoretical value, we conducted a one sample *t*-test, which found that the observed time error (0.29) is significantly smaller than the hypothesized mean ($t(47)=-17.83$, $p < 0.001$, 95% CI = [0.23,0.35]). Indicating that this strategy is a poor explanation for the observed data.

Frame 1	Frame 2	Frame 3	Frame 4	Frame 5
-4	-3	-2	**-1**	**0**
-3	-2	**-1**	**0**	**1**
-2	**-1**	**0**	**1**	**2**
**-1**	**0**	**1**	**2**	**3**
**0**	**1**	**2**	**3**	**4**

Supplementary Table 4. A schematic of all possible combinations of choices in the experiment. Each row represents a single trial where Frame 1 represents the earliest frame and Frame 5 represents the latest frame among the 5 alternatives presented. Frame order was randomized in the experiment. The value of the cells represents the frames available, expressed as the time error from the target frame (represented by 0). There is an equal proportion of trials represented by each row. Bolded frames indicate frames that could not have appeared in the preview, and were averaged in the calculation of the mean time error if participants ignored previously seen frames. Non-bolded frames would have been previously seen by the participant during video previews.

However, given that some road scenes remain quite similar on a second-to-second scale, participants may not have been able to perfect recognize already seen frames. If so, then participants may have selected previously seen frames erroneously on these trials because they were unable to distinguish them from the new frames. To partial out the effects of these guesses, we can take the subset of trials on which no prior frames were shown and examine whether the observed mean error differ from random guessing. These trials constitute 2 out of the 5 possible trial types, corresponding to the bottom two rows listed in Supplementary Table 4, resulting in 144 useable trials out of 360 total trials. A one-sample *t*-test failed to find a significant difference between the observed time error on these trials (1.43) with a mean time error that would have resulted from random guessing (1.5; $t(47) = -1.78$, $p = 0.08$, 95%*CI* = [1.35,1.51]), suggesting that the forward bias may be a result of the set-up of the task. However, given the reduced number of trials, there may not have been enough power in this subset of data to detect such a small effect. Repeating this procedure using standard deviation of time errors revealed that the observed selections are slightly more consistent across trials (1.42) than a theoretical standard deviation (1.58) using this strategy ($t(47) = -5.41$, $p < 0.001$, 95%*CI* = [1.40,1.45]), suggesting that participants were able to predict the scene to some extent and not simply randomly guessing.

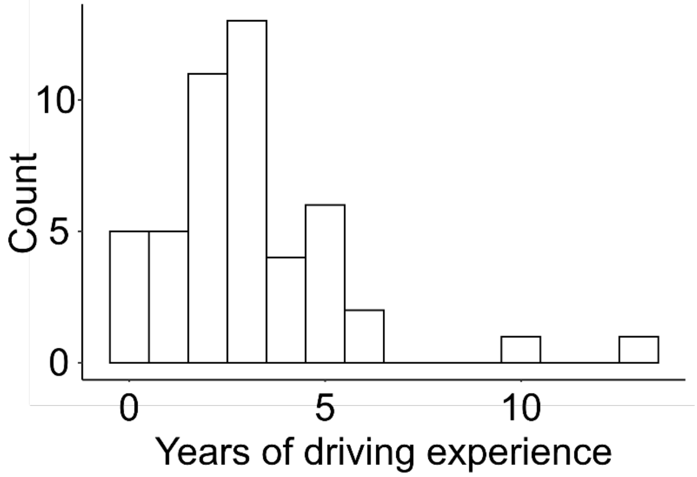

Supplementary Figure 5. A histogram of the sample's driving experience in years. Out of 48 participants, one declined to provide when they started driving and was excluded from this figure. The 5 participants who have 0 years of experience have at least 8 months of experience since that is the minimum required time from the first practical road test for obtaining a G2 in Ontario, Canada. All participants in the sample are holders of a class G2 or G license or equivalent which was verified by the experimenter at the beginning of each session.
